# Albisporachelin, a New Hydroxamate Type Siderophore from the Deep Ocean Sediment-Derived Actinomycete *Amycolatopsis* *albispora* WP1^T^

**DOI:** 10.3390/md16060199

**Published:** 2018-06-07

**Authors:** Qihao Wu, Robert W. Deering, Gaiyun Zhang, Bixia Wang, Xin Li, Jiadong Sun, Jianwei Chen, Huawei Zhang, David C. Rowley, Hong Wang

**Affiliations:** 1 College of Pharmaceutical Science, Zhejiang University of Technology, Hangzhou 310014, China; qihaowu@zjut.edu.cn (Q.W.); 15757116051@163.com (B.W.); lxzjut@126.com (X.L.); cjw983617@zjut.edu.cn (J.C.); hwzhang@zjut.edu.cn (H.Z.); 2 Department of Biomedical and Pharmaceutical Science, College of Pharmacy, University of Rhode Island, Kingston, RI 02881, USA; rdeering@oceanspray.com (R.W.D.); jiadong.sun@nih.gov (J.S.); 3 Key Laboratory of Marine Biogenetic Resources, Third Institute of Oceanography, State Oceanic Administration, Xiamen 361005, China; zhanggyun@126.com

**Keywords:** *Amycolatopsis*, siderophore, deep ocean sediment

## Abstract

Marine actinobacteria continue to be a rich source for the discovery of structurally diverse secondary metabolites. Here we present a new hydroxymate siderophore produced by *Amycolatopsis albispora*, a recently described species of this less explored actinomycete genus. Strain WP1^T^ was isolated from sediments collected at −2945 m in the Indian Ocean. The new siderophore, designated albisporachelin, was isolated from iron depleted culture broths and the structure was established by 1D and 2D NMR and MS/MS experiments, and application of a modified Marfey’s method. Albisporachelin is composed of one *N*-methylated-formylated/hydroxylated l-ornithine (*N*-Me-fh-l-Orn), one l-serine (l-Ser), one formylated/hydroxylated l-ornithine (fh-l-Orn) and a cyclo-*N*-methylated-hydroxylated l-ornithine (cyclo-*N*-Me-h-l-Orn).

## 1. Introduction

More than 70% of the earth’s surface is covered by ocean, and about 60% of the ocean floor lies beneath water more than 2000 m deep. Due to obvious technical challenges, deep ocean sediments are perhaps the largest and least known environment for microbiology. Iron is an essential element for nearly all organisms, but soluble Fe(III), predominately in the form of Fe(OH)_3_, is normally present in trace concentrations in the deep sea [1], thus creating microbial competition for this vital element. The production of chelating agents, known as siderophores, is the most common strategy employed by bacteria for scavenging iron [2,3]. These low molecular weight compounds (500–1500 daltons) can be broadly classified by the types of chelating groups that bind iron, and include catechols, α-hydroxycarboxylates, hydroxamates, ο-hydroxyphenyloxazolines, and mixed types [2,4]. While more than 500 siderophores have been reported [4], there are few representatives from deep ocean environments such as fradiamine A [5], tetroazolemycins [6], and lystabactins [7].

During the course of our natural products screening program from deep-ocean bacteria, we found that a marine sediment derived strain *Amycolatopsis albispora* WP1^T^, a proposed new species, produced siderophores under iron-deficient culture conditions. *Amycolatopsis* produce secondary metabolites with uses in medicine and agriculture, including vancomycin and rifamycin [8]. To date, only two siderophores have been identified from *Amycolatopsis* [9,10]. Hence, we were intrigued by the production of a siderophore from a newly described deep ocean species of *Amycolatopsis*, and herein report the discovery of a new marine hydroxamate siderophore, designated as albisporachelin.

## 2. Results

**Figure 1:**
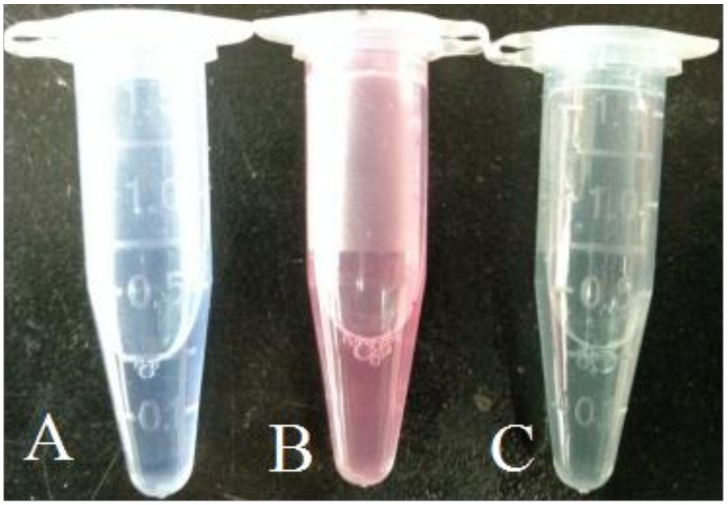
Siderophore detection of *A. albispora* WP1^T^ by liquid CAS method. A: Liquid CAS detection of *A. albispora* WP1^T^ cultivated in siderophore producing media with Fe^3+^; B: Liquid CAS detection of *A. albispora* WP1^T^ cultivated in siderophore producing media without Fe^3+^; C: Liquid CAS detection of siderophore producing media.

**Figure 2:**
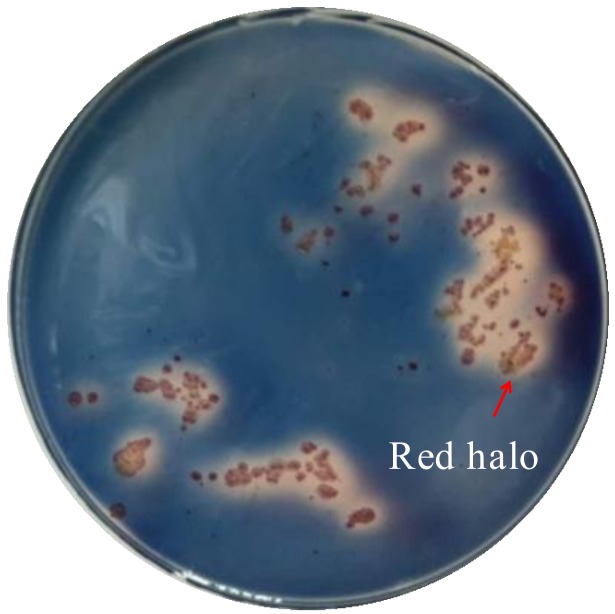
Siderophore detection of *A. albispora* WP1^T^ by CAS double-layer agar diffusion method. Red haloes occur when siderophore was produced by the tested strains.

Strain WP1^T^ was isolated from sediments collected in the Indian Ocean at a water depth of 2945 m. Strain WP1^T^ formed white mycelia on ISP_2_ agar plate with 3% sea salt at 30 °C for 5 days (Supporting Information (SI), Figure S1). Careful analysis of mycelial morphology and 16S rRNA sequence comparison resulted in the identification of this strain as an *Amycolatopsis albispora* sp. nov. (SI, Figure S2) [11]. The strain was confirmed as a siderophore producer using the modified liquid and agar plate CAS (Chrome azurol S) method (Figure 1 and Figure 2) [12]. The supernatant of *A. albispora* WP1^T^ in iron deficient culture media (50 μL) was added to 50 μL CAS solution, resulting in a red solution within five seconds, thereby indicating the presence of iron-chelating agents. Supernatant EtOH crude extract (about 300 mg) of strain WP1^T^, followed by CAS activity-guided isolation using Prep-HPLC and Semi-prep-HPLC, resulted in the isolation of 15.4 mg of compound **1** (SI, Figure S3). The structure was elucidated using combined 1D/2D NMR and MS experiments, and use of a modified Marfey’s method to define the stereochemistry.

### 2.1. Structural Identification of Albisporachelin from Amycolatopsis albispora WP1^T^

**Figure 3:**
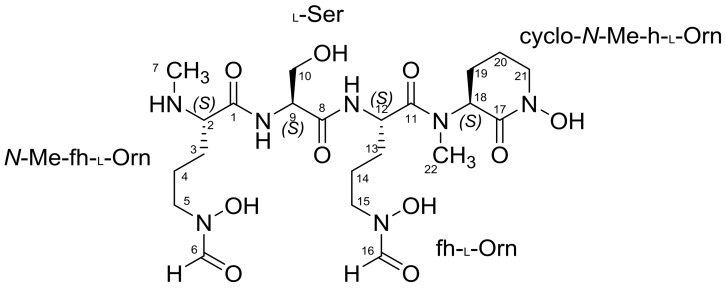
Chemical structure of albisporachelin (**1**).

Albisporachelin (**1**, Figure 3) was obtained as an optically active brown gum, (${\left[\alpha \right]}_{D}^{20}$ −16.8 (c 0.1, H_2_O)), with a molecular formula of C_22_H_39_O_10_N_7_ according to the HR-ESI-MS analysis in negative ion mode at *m/z* 560.2712 for [M − H]^−^ ((Calcd for C_22_H_38_O_10_N_7_, 560.2680), (SI, Figure S4)), corresponding to seven degrees of unsaturation. The ^1^H NMR spectral data (SI, Figure S5) showed features characteristic of a peptide, with several amide protons around 8–9 ppm, and α-protons in the range of 3–5 ppm. Resonances in the ^1^H and ^13^C NMR spectra (Table 1, Figure S6) with support from HSQC correlations indicated the presence of two formyl groups (δ_C_ 157.2, δ_H_ 7.91 and δ_C_ 161.8, δ_H_ 8.25), four amide carbonyl groups (δ_C_ 166.0, 166.6, 167.1, and 168.7), and an oxygenated carbon (δ_C_ 60.6, δ_H_ 3.56, 3.75). Absorptions in the infrared spectra (IR) indicated the presence of C=O and CHO groups (SI, Figure S7). These data combined with the degree of unsaturation suggested a single ring system in the structure of **1**.

**Figure 4:**
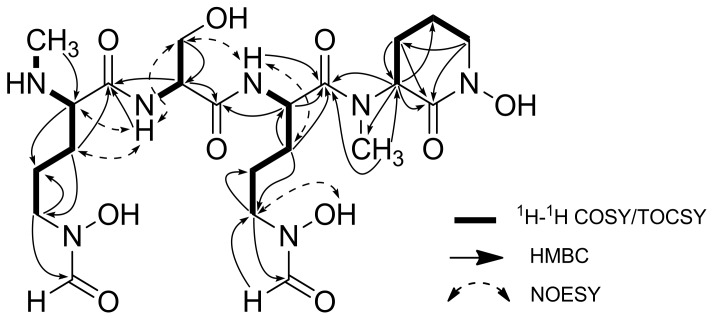
Key ^1^H-^1^H COSY, TOCSY and selected HMBC correlations of albisporachelin (**1**).

Analysis of ^1^H-^1^H COSY and TOCSY cross-peaks (SI, Figures S8 and S9) allowed the construction of proton spin systems for each amino acid (the bold bonds in Figure 4). A combination of HSQC data (SI, Figure S10) and HMBC (SI, Figure S11) correlations, including α-proton or amide proton correlations to carboxyl carbons, helped further establish the sequence of amino acids. The gCOSY and TOCSY correlations from H-7 to H-5, along with the HMBC correlations of H-5/C-6, H-7/C-2 and H-2/C-7 provided the spin system of *N*-Me-fh-Orn. A Ser residue was identified by chemical shifts of C-10 (δ_C_ 60.6) as well as the H-9/H-10; H-9/NH-2, ^1^H-^1^H spin system and the HMBC correlations of H-10 to C-9 and C-8. The HMBC cross-peaks of H-9/C-1 and NH-2/C-1 connected the Ser to the *N*-Me-fh-Orn. The gCOSY and TOCSY correlations from H-12 to H-13/H-14/H-15 and the HMBC correlation between H-16/C-15 and H-15/C-16 were used to establish the connectivity of the fh-Orn residue, and an H-12/C-8 HMBC provided connection to the Ser. Based on the unsaturation calculation, gCOSY and HMBC correlations, the final residue was determined to be a six-membered ring. The existence of an *N*-methyl group was confirmed by an HMBC correlation from H-22/C-18 and H-18/C-22. The H-18/C-17 and H-21/C-17 HMBC correlations completed the cyclo-*N*-Me-hOrn. An HMBC correlation from α-proton (δ_H_ 3.86) and *N*-methyl (δ_H_ 2.81) to the fh-Orn carbonyl carbon (δ_C_ 166.0) established its position in the molecule.

**Figure 5:**
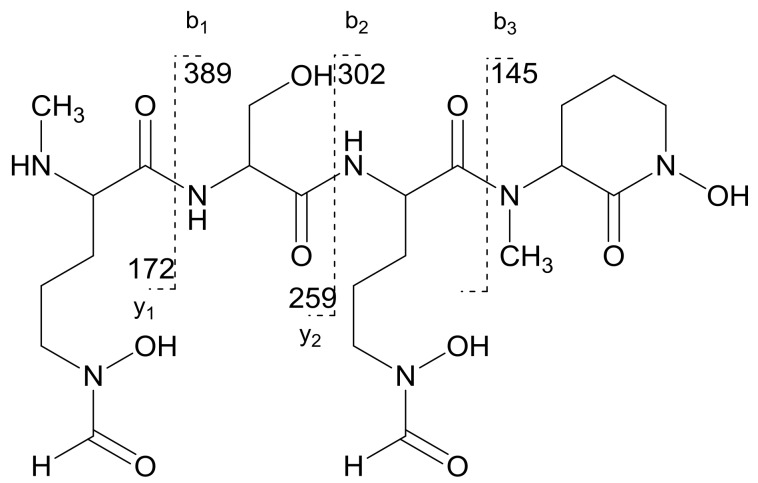
Product ions observed during MS^2^ fragmentation experiments and assignment of the molecular ion peak.

NOESY spectral data (SI, Figure S12) showed a correlation between the α-proton (δ_H_ 2.81) of *N*-Me-fh-Orn and amide proton (δ_H_ 8.76) of Ser, further establishing the connection between *N*-Me-fh-Orn and Ser. A NOESY correlation between the Ser H-10 (δ_H_ 2.56, 2.74) and amide proton (δ_H_ 8.39) of the fh-Orn residue also was consistent with the proposed structure. NOESY correlations also helped establish the hydroxyl residues in Ser (δ_H_ 10.07) and fh-Orn (δ_H_ 6.60). The attachment of other two hydroxyl groups were supported by ESI-MS/MS fragmentation patterns (Figure 5). The ESI-MS/MS of albisporachelin yield ions with *m*/*z* 390 ([M + H − 172]^+^), *m*/*z* 303 ([M + H − 172 − 87]^+^), *m*/*z* 145 ([M + H − 172 − 87 − 159]^+^). The losses of 172, 87, and 159 are assigned to the loss of *N*-Me-fh-Orn, serine, and fh-Orn, respectively (Figure 5; Figures S13 and S14). 

With the planar structure established, the configurations of the four chiral centers were determined using a modified Marfey’s method [13]. Albisporachelin was hydrolyzed with HI (Hydrogen iodide) and the resulting amino acids were derivatized with *N*α-(5-fluoro-2,4-dinitrophenyl)-l-alaninamide (l-FDAA). Hydrolysis with HI provides the additional advantage of reducing hydroxylamines [7,10,14]. The derivatized hydrolysates were then compared with amino acid standards derivatized with l-FDAA or d-FDAA using LC/MS (Figures S15–S21). Accordingly, albisporachelin was determined to be composed of l-*N*-Me-fh-Orn, l-Ser, l-fh-Orn and l-cyclo-*N*-Me-h-Orn.

### 2.2. Iron-Chelating Ability of Albisporachelin (1)

Albisporachelin (0.1 mg) was dissolved in 1 mL deionized water, and 0.05 mL 1 M FeCl_3_ was added to the solution to convert unbound albisporachelin into the corresponding ferric complex. The resulting complex was analyzed by HPLC coupled with a PDA (photo-diode array) detector. The UV spectroscopic analysis of ferric-albisporachelin complex indicates that the typical hydroxamate coordinating groups are presented in the complex, implied by the broad absorption band at about 440 nm (SI, Figure S22). These results indicated that the hydroxamate moieties are involved in iron binding [7], which was in accordance with the albisporachelin structure.

## 3. Discussion

The genus *Amycolatopsis* belongs to the family Pseudonocardiaceae and contains more than 60 defined species (http://www.bacterio. net/amycolatopsis.html) of non-motile, aerobic actinomycetes. The species have been isolated from diverse environments such as soil [15], plants [16] and clinical material [17]. WP1^T^, isolated from the sediments in the deep Indian Ocean, forms a distinct phyletic lineage in the genus *Amycolatopsis* and is a proposed new species [11]. Production of albisporachelin suggests a mechanism for acquiring iron under limited conditions, such as those found in the deep ocean.

To date, two other siderophores, amychelin and albachelin, have been identified from *Amycolatopsis* species [9,10]. In common with albisporachelin, all have a cyclized hydroxyornithine at the *C*-terminus, at least one *N*-δ-OH-*N*-δ-formyl-Orn and include serine as the only other amino acid residue. Amychelin is distinguished by the inclusion of a 2-hydroxybenzoyl-oxazoline moiety as a third chelating group. Amychelin is biosynthesized by a non-ribosomal peptide synthetase (NRPS), and a putative NRPS has been identified for albachelin biosynthesis [9,10]. A complete genome for *A. albispora* WP1^T^ is not yet available for identification of its biosynthetic gene cluster. Albisporachelin has the lowest molecular weight of the three, and therefore it is perhaps more economical to biosynthesize in the deep ocean where nutrients are low and growth rates are typically very slow. However, this hypothesis remains to be explored.

The three ornithine hydroxymates serve as the chelating ligands for coordinating iron, thus comprising a hexadentate hydroxamate siderophore commonly known to coordinate iron in an octahedral geometry [4]. Albisporachelin further shares structural similarities with other tris-hydroxymate siderophores, such as scabichelin and turgichelin produced by a *Streptomyces scabies* 87.22† [18]. However, unlike scabichelin and turgichelin, all amino acids in albisporachelin have the l-configuration.

The discovery of albisporachelin adds to the diversity of marine-derived siderophores and furthermore suggests an impact on marine chemistry, biogeochemistry and marine ecology. Iron in the ocean is mostly supplied by atmospheric dust deposition, release from sediments and deep sea hydrothermal activity [19,20]. Deep-sea hydrothermal venting fluxes 1000–10,000 Gg of soluble Fe annually to the bottom of the ocean but the majority precipitates rapidly as biologically unavailable Fe sulfide or an oxide mineral form [21]. Investigation by Li et al. has shown that microbial iron uptake and stabilization via formation of Fe-organic compound complexes is a major way of dispersing the bioavailable iron from thermal venting into the circulation of the ocean [21]. Metatranscriptomic data on a bacterial community within deep-sea hydrothermal plumes shows an enrichment of siderophore related genes, suggesting an important role for deep-sea bacterial growth and a widely adopted mechanism across the whole of deep-sea bacterial communities. However, deep-sea derived siderophores are less explored and our report here represents one of the few examples to date.

## 4. Materials and Methods

### 4.1. General Experimental Procedures

All reagents and solvents were used as received from commercial suppliers (Sigma-Aldrich, St. Louis, MO, USA). IR spectra were acquired with a Nicolet 380 FT-IR (Thermo Fisher Scientific, Waltham, MA, USA) equipped with a ZnSe ATR plate. NMR experiments were conducted using a 500 MHz Varian Inova NMR spectrometer (Agilent, Santa Clara, CA, USA) with (CD_3_)_2_SO as the solvent (referenced to residual DMSO at δ_H_ 2.54 and δ_C_ 39.5) at 25 °C. Electrospray ionization mass spectra (ESI-MS) and MS/MS were acquired using an AB Sciex TripleTOF 4600 spectrometer (AB Sciex, Framingham, MA, USA). Optical rotation was measured on a Jasco P-2000 digital polarimeter (Jasco, Tokyo, Japan) with a sodium lamp (589 nm). The preparative HPLC was performed on a Shimadzu LC-6AD HPLC system (Shimadzu, Nakagyo-ku, Kyoto, Japan) with a Phenomenex Luna C18 5 µm, 21.20 mm × 250 mm column (Torrance, CA, USA). The semi-preparative HPLC was performed on a Shimadzu LC-2030C HPLC system (Shimadzu, Nakagyo-ku, Kyoto, Japan) with a Waters Xbridge prep C18 5 μm, 10 mm × 250 mm column (Waters, Milford, MA, USA). LC-MS was carried out on an AB Sciex QTRAP 4500 (AB Sciex, Framingham, MA, USA). 

### 4.2. Bacteria Strain

The isolate *A. albispora* WP1^T^ was isolated from a deep-sea sediment sample collected from the Indian Ocean at site TVG1 (27.9° S 63.5° E; −2945 m). The isolation media was modified Zobell 2216E agar. Strain WP1^T^ was identified as a Gram-positive actinomycete and formed well-developed branched substrate mycelium as well as profuse aerial mycelium on MZ2 medium [11]. The strain was identified by morphology and 16S rRNA gene sequence analysis and was deposited in the China General Microbiological Culture Collection Centre (CGMCC), Beijing, China, under the accession number CGMCC 10738.

### 4.3. Siderophore Screening

CAS solution was prepared as previously reported [12]. Test samples (50 μL) and CAS solution (50 μL) were combined in wells of a polystyrene 96-well plate. Iron-complexing metabolites were detected by a color change from blue to red in under 5 s.

### 4.4. Fermentation, Extraction and Isolation

*A. albispora* WP1^T^ was cultured in shaking incubators at 180 rpm and 30 °C for 5 days using 1 L polypropylene flasks containing 400 mL of iron deficient culture medium, which was prepared as follows. Two g of K_2_SO_4_, 3 g of K_2_HPO_4_, 1 g of NaCl and 5 g of NH_4_Cl were dissolved in 1 L of deionized water. To remove ferric ions, the solution was stirred with 50 g of chelex-100 Na^+^ form (Bio-Rad, Hercules, CA, USA) for 2 h. The solution was filtered through Whatman No. 1 filter paper, and 10 mL of each of the following sterile solutions were added to the medium immediately prior to use: CaCl_2_·H_2_O (10 mg/mL), glucose (250 mg/mL), and 0.5% yeast extract (Difco) [15]. The culture broth (a total of 2 L) was concentrated to dryness in vacuo and extracted with 1 L of EtOH [22]. The EtOH extract was concentrated in vacuo, re-dissolved in 100 mL DI water and passed through a 0.22 μm filter. The solution was fractionated on a Shimadzu LC 6AD equipped with a Phenomenex preparative column (Torrance, CA, USA, Luna 5 µm C18, 21.20 mm × 250 mm) with isocratic elution of 95% H_2_O with 0.1% TFA and 5% acetonitrile. CAS positive fractions were combined to afford a crude sample (30 mg). The crude sample was then subjected to Shimadzu LC-2030C on Waters XBridge Prep C18 column (5 μm, 10 mm × 250 mm) with gradient elution of H_2_O/MeOH (from 98:2 to 90:10 in 20 min) containing 0.1% formic acid. A total of 15.4 mg albisporachelin was obtained.

### 4.5. Acid Hydrolysis of Compound ***1*** and Assignment of the Absolute Configuration by Modified Marfey’s Method

Albisporachelin (0.2 mg) was hydrolyzed at 110 °C for 12 h with 50% HI solution (0.5 mL). The hydrolysate was dried by rotary evaporator and dissolved in H_2_O (200 μL). To derivatize, 200 μL of a solution of l-FDAA or d-FDAA (1% *w*/*v* in acetone) and 80 μL of 1 M NaHCO_3_ was added to the aliquot of the hydrolysate, after which the mixtures were stirred and heated at 50 °C for 1 h. The reaction mixtures were neutralized by addition of 1 N HCl (80 μL) after cooling to room temperature, then concentrated to dryness and re-suspended in MeOH (200 μL). Derivatives were analyzed by HPLC on a C18 column (Waters XBridge, 4.6 mm × 50 mm) with PDA detection monitoring absorbances from 190 to 600 nm. The HPLC analysis was performed using a linear gradient of 5–25% MeCN in H_2_O (each with 0.05% TFA) over 60 min at a flow rate of 1 mL/min. The retention times (min) of the l-and d-FDAA derivatized amino acids were l-Ser-l-FDAA (50.0 min), l-Ser-d-FDAA (48.5 min), l-Orn-l-FDAA (37.4 min), l-Orn-d-FDAA (35.1 min), l-*N*-Me Orn-l-FDAA (35.6 min), and l-*N*-Me Orn-d-FDAA (33.4 min). The albisporachelin hydrolysate and AA standards were further subjected to HPLC-MS analysis. LC/MS analysis was performed using a linear gradient of 5–25% MeCN in H_2_O (each with 0.1% FA) over 50 min at flow rate of 0.5 mL/min.

## Figures and Tables

**Table 1:** ^1^H-(500 MHz) and ^13^C-NMR (125 MHz) data of **1** in DMSO-*d6* (δ in ppm, *J* in Hz).

Residue	Position	δ_C_, Type	δ_H_ (J in Hz)	^1^H-^1^H COSY	HMBC
*N*-Me-hf-Orn	C-1	167.1, qC	-	-	-
	C-2	60.2, CH	3.86 m	NH-1, H-3	C-1, C-3, C-4
	C-3	32.8, CH_2_	1.54, 1.72 m	H-2, H-4	C-1, C-2, C-4, C-5
	C-4	22.6, CH_2_	1.62 m	H-3, H-5	C-2, C-3, C-5
	C-5	48.7, CH_2_	3.43 m	H-4	C-3, C-4, C-6
	C-6	157.2, CH	7.90 d (8.5)	-	-
	C-7	31.3, CH_3_	2.48 s	-	C-2
	NH-1	-	8.87 s	-	-
Ser	C-8	168.7, qC	-	-	-
	C-9	52.8, CH	5.04 m	H-10	C-1, C-8, C-10
	C-10	60.6, CH_2_	3.56, 3.75 m	H-9	C-8, C-9
	NH-2	-	8.79 d (7.6)	H-9	C-1
Hf-Orn	C-11	166.0, qC	-	-	-
	C-12	54.3, CH	3.75 m	NH-3, H-13	C-8, C-11
	C-13	32.4, CH_2_	1.54, 1.72 m	H-12, H-14	C-11, C-12, C-15
	C-14	26.5, CH_2_	1.72 m	H-13, H-15	C-12, C-13, C-15
	C-15	45.5, CH_2_	3.43 m	H-14	C-13, C-14, C-16
	C-16	161.8, CH	8.24 d (6.9)	-	C-15
	NH-3	-	8.37 d (17.5)	-	-
Cyclic *N*-Me-fh-Orn	C-17	166.6, qC	-	-	-
	C-18	60.4, CH	3.85 m	H-19	C-17, C-19, C-20, C-22
	C-19	28.8, CH_2_	1.63, 1.81 m	H-18, H-20	C-17, C-18, C-20
	C-20	21.9, CH_2_	1.63 m	H-19, H-21	C-18, C-19, C-21
	C-21	47.1, CH_2_	3.73, 3.37 m	H-20	C-17, C-19, C-20
	C-22	31.9, CH_3_	2.81 s	-	C-11, C-18

## Supplementary Materials

The following are available online at http://www.mdpi.com/1660-3397/16/6/199/s1, Figure S1: Colony morphology of *A. albispora* WP1^T^ on ISP_2_ agar plate, Figure S2: The 16S rRNA phylogram of representative *Amycolatopsis* strains, Figure S3: CAS activity-guided isolation of siderophore from the supernatant extract of *A. albispora* WP1^T^, Figure S4: HR-ESI-MS spectrum of albisporachelin in negative mode, Figure S5: ^1^H NMR spectrum of albisporachelin in DMSO-*d6*, Figure S6: ^13^C NMR spectrum of albisporachelin in DMSO-d6, Figure S7: FT-IR-spectrum of albisporachelin, Figure S8: ^1^H-^1^H COSY spectrum of albisporachelin in DMSO-*d6*, Figure S9: TOCSY spectrum of albisporachelin in DMSO-*d6*, Figure S10: HSQC spectrum of albisporachelin in DMSO-*d6*, Figure S11: HMBC spectrum of albisporachelin in DMSO-*d6*, Figure S12: NOESY spectrum of albisporachelin in DMSO-*d6*, Figure S13: Observed main fragments list of compound **1** during MS^2^ fragmentation experiments, Figure S14: Main fragments observed during MS^2^ fragmentation experiments and assignment of the molecular ion peak, Figure S15: HPLC trace of FDAA-derivatized albisporachelin hydrolysate with standard, Figure S16: LC-MS-traces of FDAA-derivatized albisporachelin hydrolysate, Figure S17: MS spectrum of mono-α*N*-Me-l-Orn-l-FDAA in albisporachelin hydrolysate, Figure S18: MS spectrum of mono-αl-Orn-l-FDAA in albisporachelin hydrolysate, Figure S19: MS spectrum of mono-ωl-Orn-l-FDAA in albisporachelin hydrolysate, Figure S20: MS spectrum of mono-ω*N*-Me-l-Orn-l-FDAA in albisporachelin hydrolysate, Figure S21: MS spectrum of l-Ser-l-FDAA in albisporachelin hydrolysate, Figure S22: UV-visible spectra of albisporachelin and ferric complex of albisporachelin.

## References

[1] Liu X., Milero F.J. (2002). The solubility of iron in seawater. Mar. Chem..

[2] Butler A., Theisen R.M. (2010). Iron (III)-siderophore coordination chemistry: Reactivity of marine sideophores. Coord. Chem. Rev..

[3] De Carvalho C.C., Fernandes P. (2010). Production of metabolites as bacterial responses to the marine environment. Mar. Drugs.

[4] Hider R.C., Kong X. (2010). Chemistry and biology of siderophores. Nat. Prod. Rep..

[5] Takehana Y., Umekita M., Hatano M., Kato C., Sawa R., Igarashi M. (2017). Fradiamine A, a new siderophore from the deep-sea actinomycete *Streptomyces fradiae* MM456M-mF7. J. Antibiot..

[6] Liu N., Shang F., Xi L., Huang Y. (2013). Tetroazolemycins A and B, two new oxazole-thiazole siderophores from deep-sea *Streptomyces olivaceus* FXJ8.012. Mar. Drugs.

[7] Zane H.K., Butler A. (2013). Isolation, structure elucidation, and iron-binding properties of lystabactins, siderophores isolated from a marine *Pseudoalteromonas* sp.. J. Nat. Prod..

[8] Chen S., Wu Q., Shen Q., Wang H. (2016). Progress in understanding the genetic information and biosynthetic pathways behind *Amycolatopsis* antibiotics, with implications for the continue discovery of novel drugs. ChemBioChem.

[9] Seyedsayamdost M.R., Traxler M.F., Zheng S., Kolter R., Clardy J. (2011). Structure and biosynthesis of amychelin, an unusual mixed-ligand siderophore from *Amycolatopsis* sp. AA4. J. Am. Chem. Soc..

[10] Kodani S., Komaki H., Suzuki M., Hemmi H., Ohnishi-Kameyama M. (2015). Isolation and structure determination of new siderophore albachelin from *Amycolatopsis alba*. Biometals.

[11] Zhang G., Wang L., Li J., Zhou Y. (2016). *Amycolatopsis albispora* sp. nov., isolated from deep-sea sediment. Int. J. Syst. Evol. Microbiol..

[12] Milagres A.M.F., Machuca A., Napoleao D. (1999). Detection of siderophore production from several fungi and bacteria by a modification of chrome azurol S (CAS) agar plate assay. J. Microbiol. Methods.

[13] Bhushan R., Bruckner H. (2004). Marfey’s reagent for chiral amino acid analysis: A review. Amino Acids.

[14] Sharman G.J., Williams D.H., Ewing D.F., Ratledge C. (1995). Determination of the structure of exochelin MN, the extracellular siderophore from *Mycobacterium neoaurum*. Chem. Biol..

[15] Zucchi T.D., Tan G.Y., Bonda A.N., Frank S., Kshetrimayum J.D., Goodfellow M. (2012). *Amycolatopsis granulosa* sp. nov. *Amycolatopsis ruanii* sp. nov. and *Amycolatopsis thermalba* sp. nov. thermophilic actinomycetes isolated from arid soils. Int. J. Syst. Evol. Microbiol..

[16] Miao Q., Qin S., Bian G.K., Yuan B., Xing K., Zhang Y.J., Li Q., Tang S.K., Li W.J., Jiang J.H. (2011). *Amycolatopsis endophytica* sp. nov. a novel endophytic actinomycete isolated from oil-seed plant *Jatropha curcas* L.. Antonie Leeuwenhoek.

[17] Labeda D.P., Donahue J.M., Williams N.M., Sells S.F., Henton M.M. (2003). *Amycolatopsis kentuckyensis* sp. nov. *Amycolatopsis lexingtonensis* sp. nov. and *Amycolatopsis pretoriensis* sp. nov. isolated from equine placentas.. Int. J. Syst. Evol. Microbiol..

[18] Kodani S., Bicz J., Song L., Deeth R.J., Ohnishi-Kameyama M., Yoshida M., Ochi K., Challis G.L. (2013). Structure and biosynthesis of scabichelin, a novel tris-hydroxamate siderophore produced by the plant pathogen *Streptomyces scabies* 87.22. Org. Biomol. Chem..

[19] Boyd P.W., Ellwood M.J. (2010). The biogeochemical cycle of iron in the ocean. Nat. Geosci..

[20] Boiteau R.M., Mende D.R., Hawco N.J., McIlvin M.R., Fitzsimmons J.N., Saito M.A., Sedwick P.N., DeLong E.F., Repeta D.J. (2016). Siderophore-based microbial adaptations to iron scarcity across the eastern Pacific Ocean. Proc. Natl. Acad. Sci. USA.

[21] Li M., Toner B.M., Baker B.J., Breier J.A., Sheik C.S., Dick G.J. (2014). Microbial iron uptake as a mechanism for dispersing iron from deep-sea hydrothermal vents. Nat. Commun..

[22] Kishimoto S., Nishimura S., Hattori A., Tsujimoto M., Hatano M., Igarashi M., Kakeya H. (2014). Chlorocatechelins A and B from *Streptomyces* sp.: New siderophores containing chlorinated catecholate groups and an acylguanidine structure. Org. Lett..

