# Supplementary material for: Albisporachelin, a New Hydroxamate Type Siderophore from the Deep Ocean Sediment-Derived Actinomycete Amycolatopsis albispora WP1T
Source: Mar Drugs. 2018 Jun 7;16(6):199. doi: 10.3390/md16060199 (PMC6024995; doi:10.3390/md16060199)
Supplement: Supplementary file 1 [file marinedrugs-16-00199-s001.pdf]

Albisporachelin, a New Hydroxamate Type Siderophore from the Deep Ocean Sediment-Derived Actinomycete *Amycolatopsis albispora* WP1^T

Qihao Wu [1,§], Robert W. Deering [2,§], Gaiyun Zhang [3], Bixia Wang [1], Xin Li [1], Jiadong Sun [2], Jianwei Chen [1], Huawei Zhang [1], David C. Rowley [2,*] and Hong Wang [1,*]

1 College of Pharmaceutical Science, Zhejiang University of Technology, Hangzhou 310014, Zhejiang, China; hongw@zjut.edu.cn
2 Department of Biomedical and Pharmaceutical Science, College of Pharmacy, University of Rhode Island, Kingston, Rhode Island 02881, United States; drowley@uri.edu
3 Key Laboratory of Marine Biogenetic Resources, Third Institute of Oceanography, State Oceanic Administration, Xiamen 361005, Fujian, China.
* Correspondence: hongw@zjut.edu.cn; Tel.: +86-571-8832-0622; drowley@uri.edu; Tel.: 401-874-9228
† These authors contributed equally to this work.

Table of contents

Figures

Figure S1. Colony morphology of *A. albispora* WP1^T on ISP_2 agar plate

Figure S2. The 16S rRNA phylogram of representative *Amycolatopsis* strains

Figure S3. CAS activity guided isolation of siderophore from the supernatant extract of *A. albispora* WP1^T

Figure S4. HR-ESI-MS spectrum of albisporachelin in negative mode

Figure S5. ^{1}H NMR spectrum of albisporachelin in DMSO-*d6*

Figure S6. ^{13}C NMR spectrum of albisporachelin in DMSO-*d6*

Figure S7. FT-IR-spectrum of albisporachelin

Figure S8. ^{1}H- ^{1}H COSY spectrum of albisporachelin in DMSO-*d6*

Figure S9. TOCSY spectrum of albisporachelin in DMSO-*d6*

Figure S10. HSQC spectrum of albisporachelin in DMSO-*d6*

Figure S11. HMBC spectrum of albisporachelin in DMSO-*d6*

Figure S12. NOESY spectrum of albisporachelin in DMSO-*d6*

Figure S13. Observed main fragments list of compound **1** during MS^2 fragmentation experiments

Figure S14. Main fragments observed during MS^2 fragmentation experiments and assignment of the molecular ion peak

Figure S15. HPLC trace of FDAA-derivatized albisporachelin hydrolysate with standard

Figure S16. LC-MS-traces of FDAA-derivatized albisporachelin hydrolysate

Figure S17. MS spectrum of mono-αN-Me-L-Orn-L-FDAA in albisporachelin hydrolysate

Figure S18. MS spectrum of mono-αL-Orn-L-FDAA in albisporachelin hydrolysate

Figure S19. MS spectrum of mono-ωL-Orn-L-FDAA in albisporachelin hydrolysate

Figure S20. MS spectrum of mono-ωN-Me-L-Orn-L-FDAA in albisporachelin hydrolysate

Figure S21. MS spectrum of L-Ser-L-FDAA in albisporachelin hydrolysate

Figure S22. UV-visible spectra of albisporachelin and ferric complex of albisporachelin

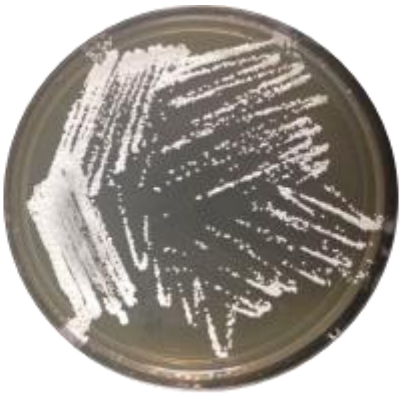

Figure S1. Colony morphology of A. albispora WP1^{T} on ISP$_2$ agar plate.

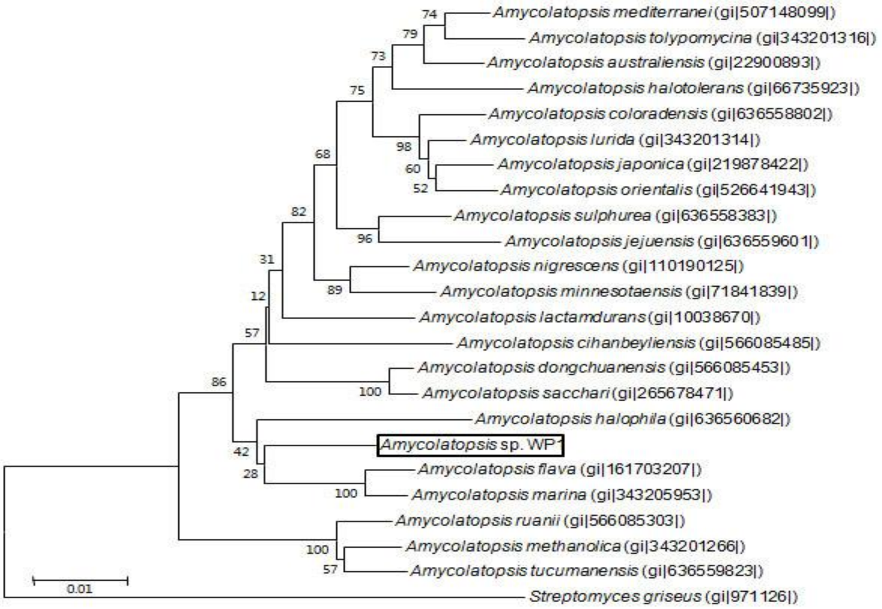

Figure S2. The 16S rRNA phylogram of representative Amycolatopsis strains. With an outgroup containing S. griseus. Ribosomal RNA sequences for all strains but A. albispora WP1^{T} were obtained from GenBank database.

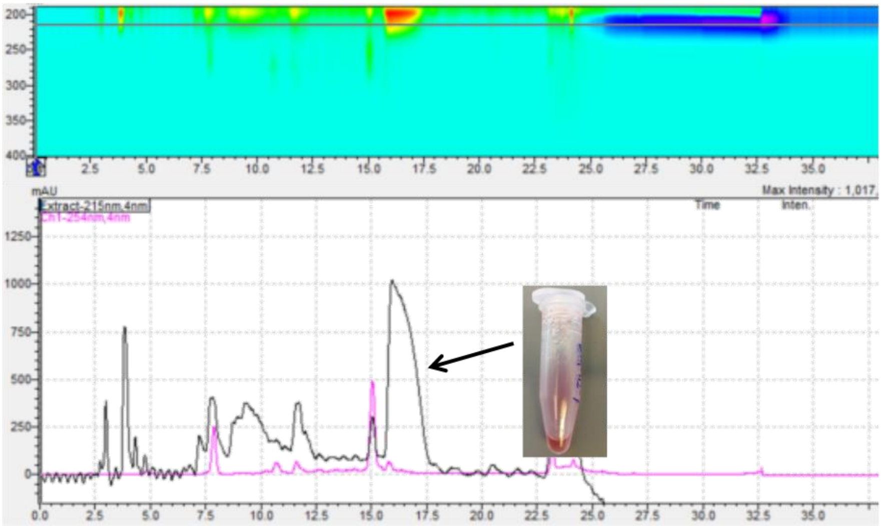

Figure S3. CAS activity guided isolation of siderophore from the supernatant extract of A. albispora WP1$^{T.}$

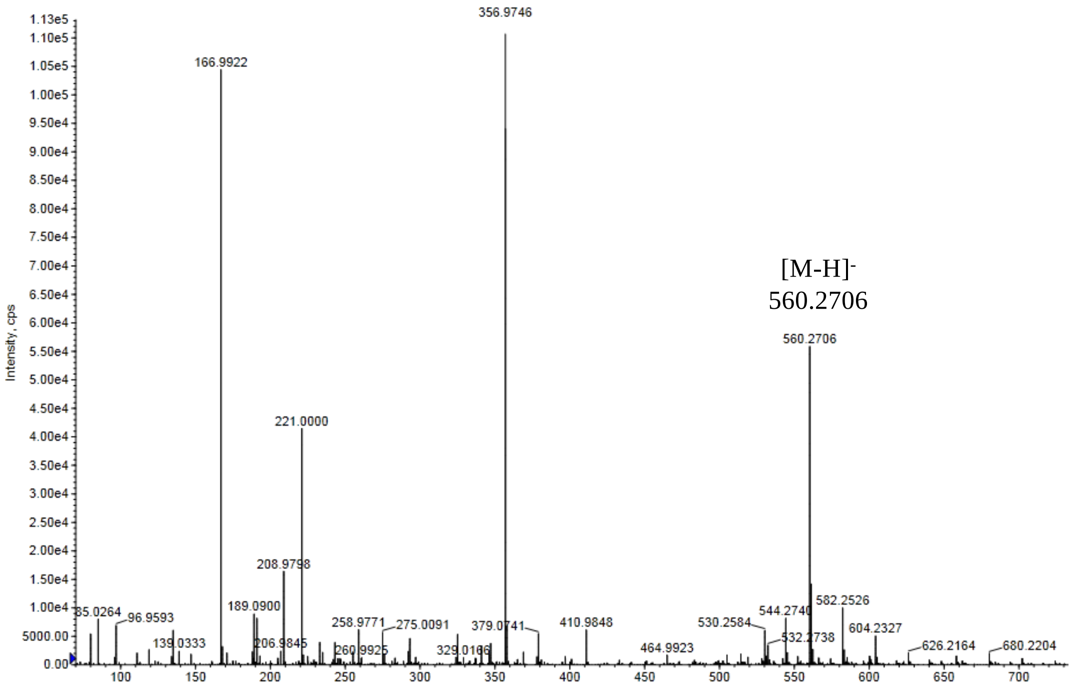

Figure S4. HR-ESI-MS spectrum of albisporachelin in negative mode.

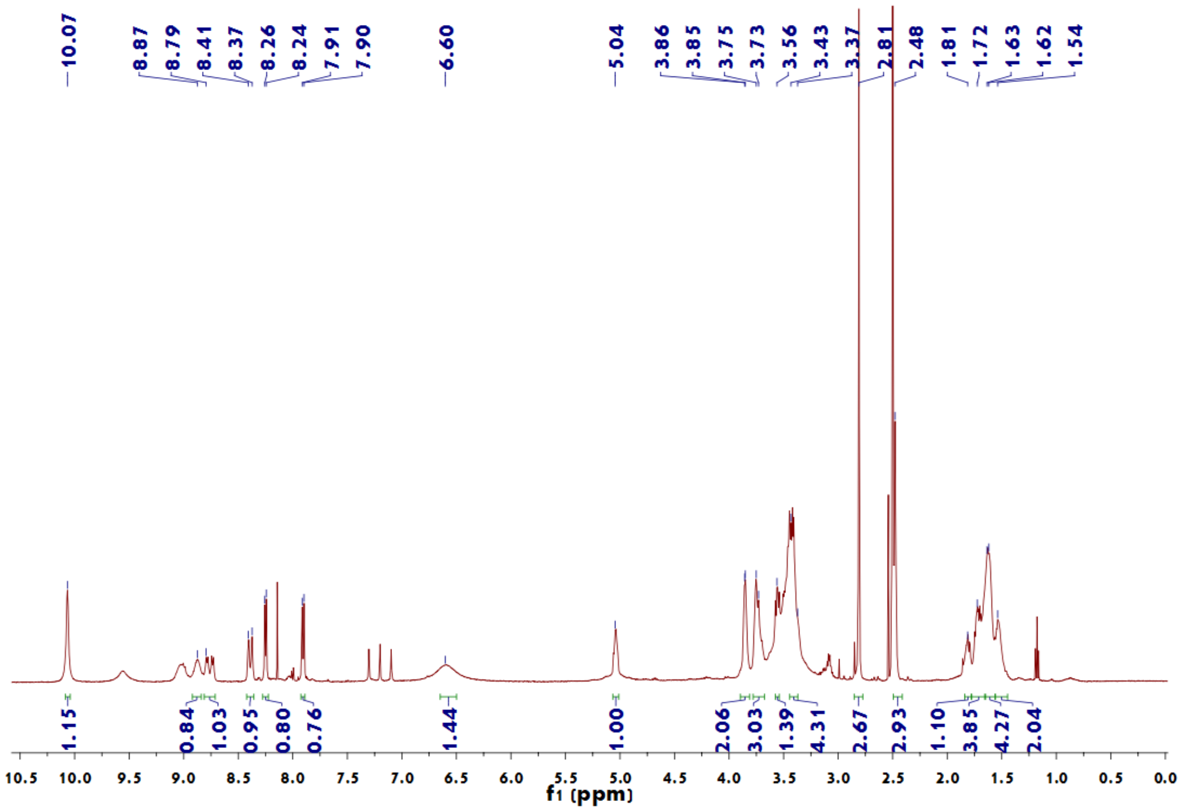

Figure S5. ^{1}H NMR spectrum of albisporachelin in DMSO-*d6*.

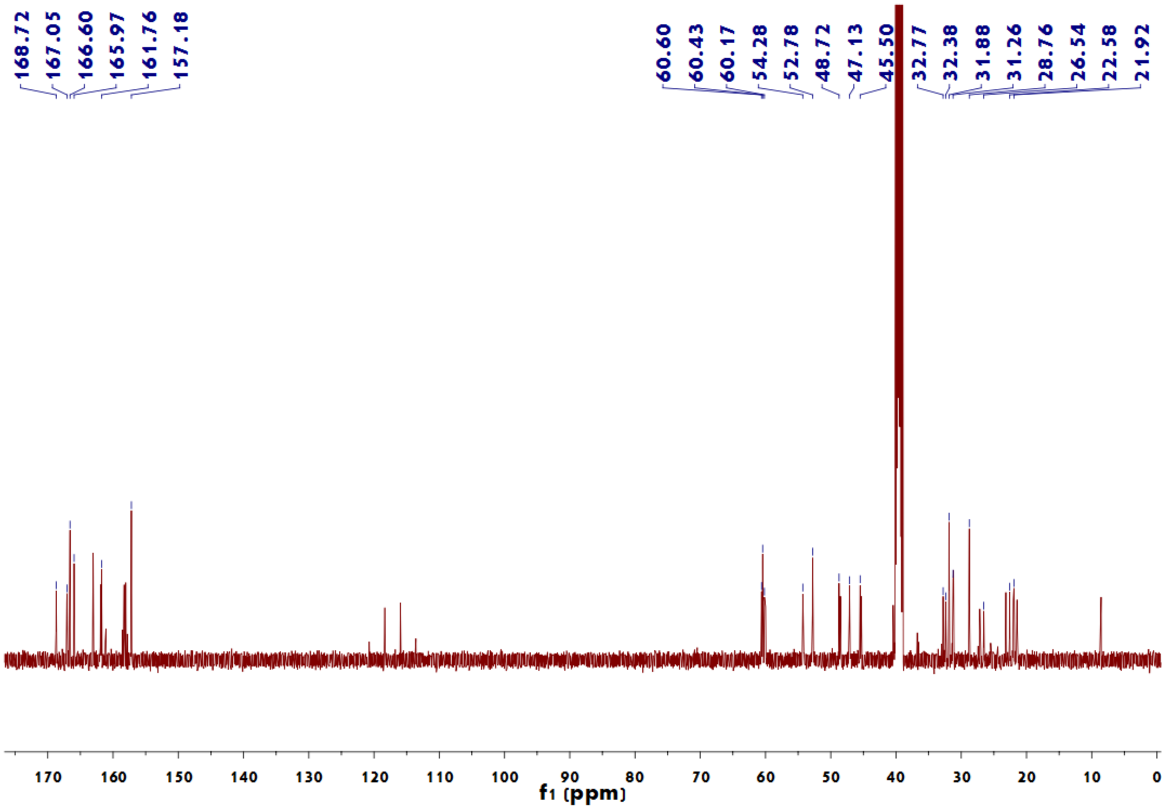

Figure S6. ^{13}C NMR spectrum of albisporachelin in DMSO-*d6*.

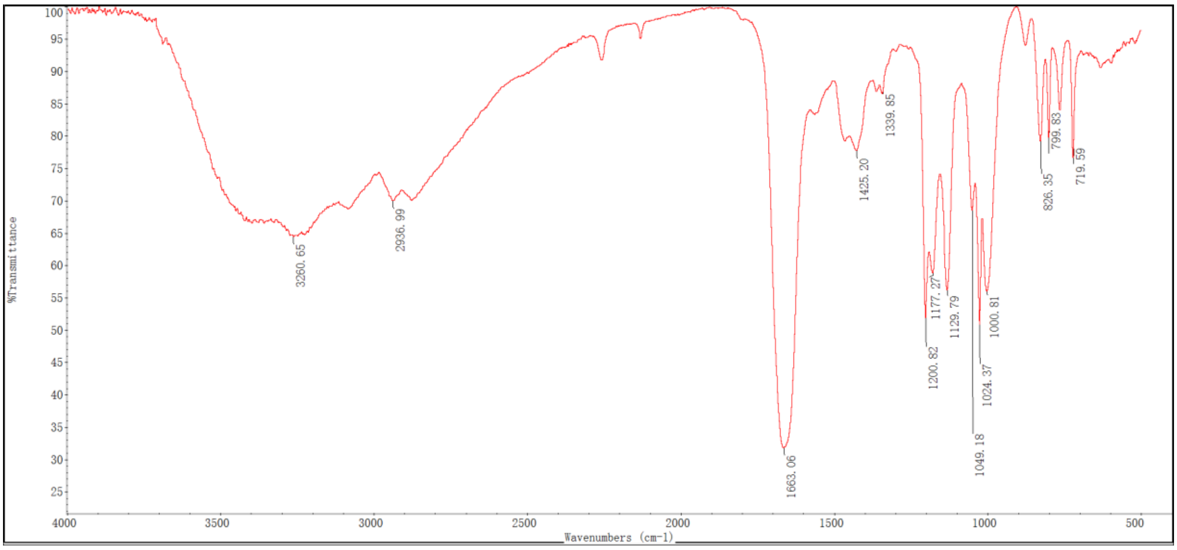

Figure S7. FT-IR-spectrum of albisporachelin.

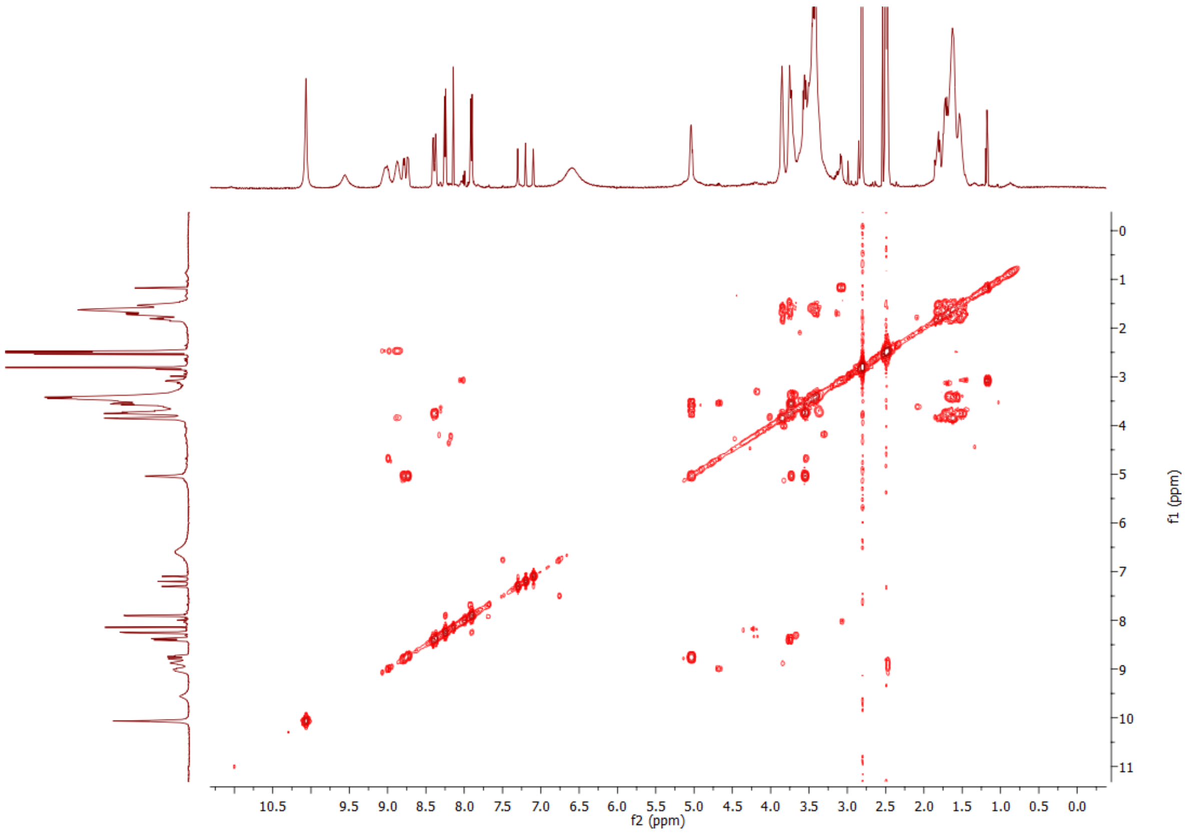

Figure S8. ^{1}H- ^{1}H COSY spectrum of albisporachelin in DMSO-*d6*.

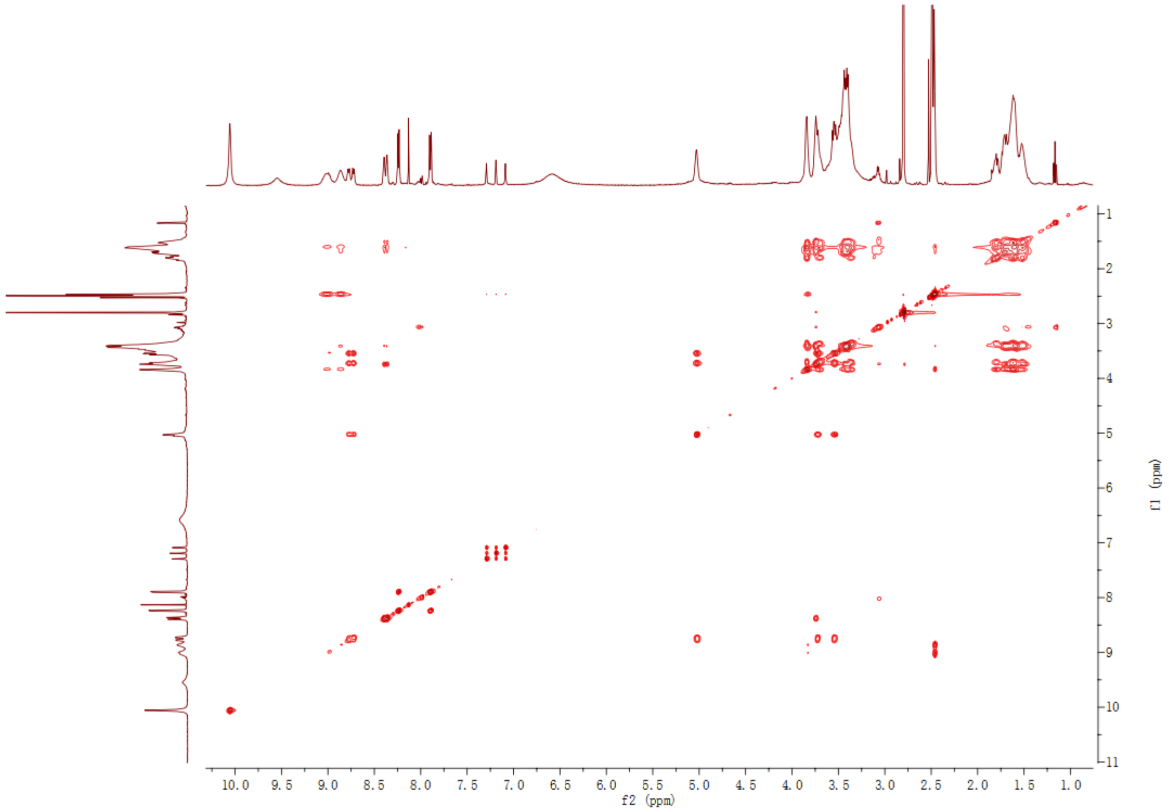

Figure S9. TOCSY spectrum of albisporachelin in DMSO-*d6*.

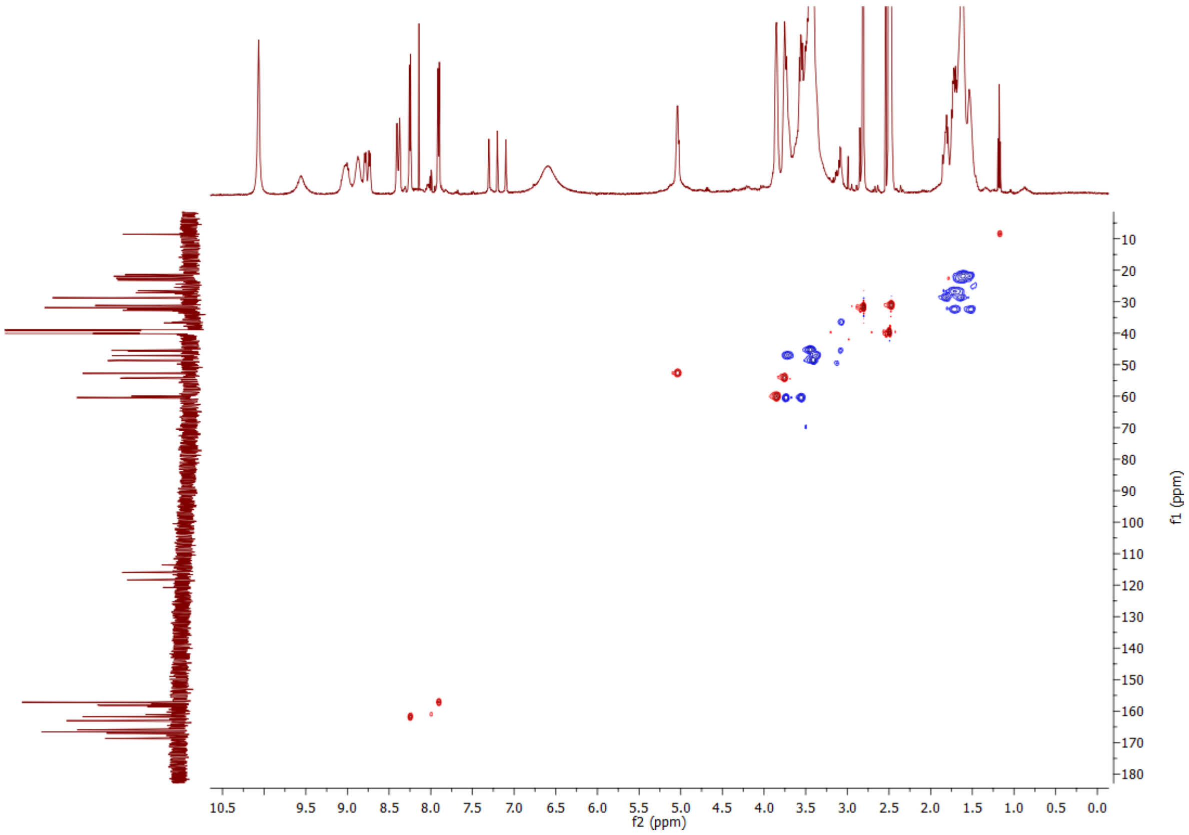

Figure S10. HSQC spectrum of albisporachelin in DMSO-*d6*.

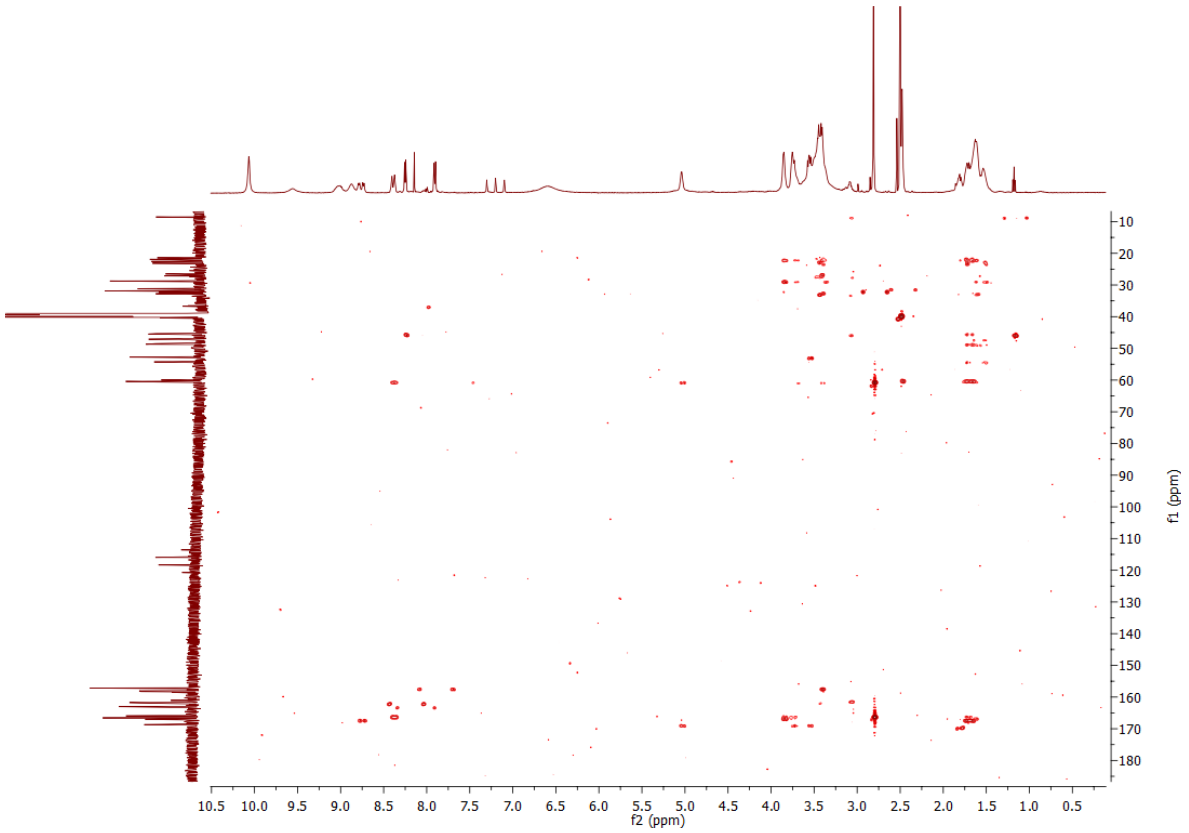

Figure S11. HMBC spectrum of albisporachelin in DMSO-*d6*.

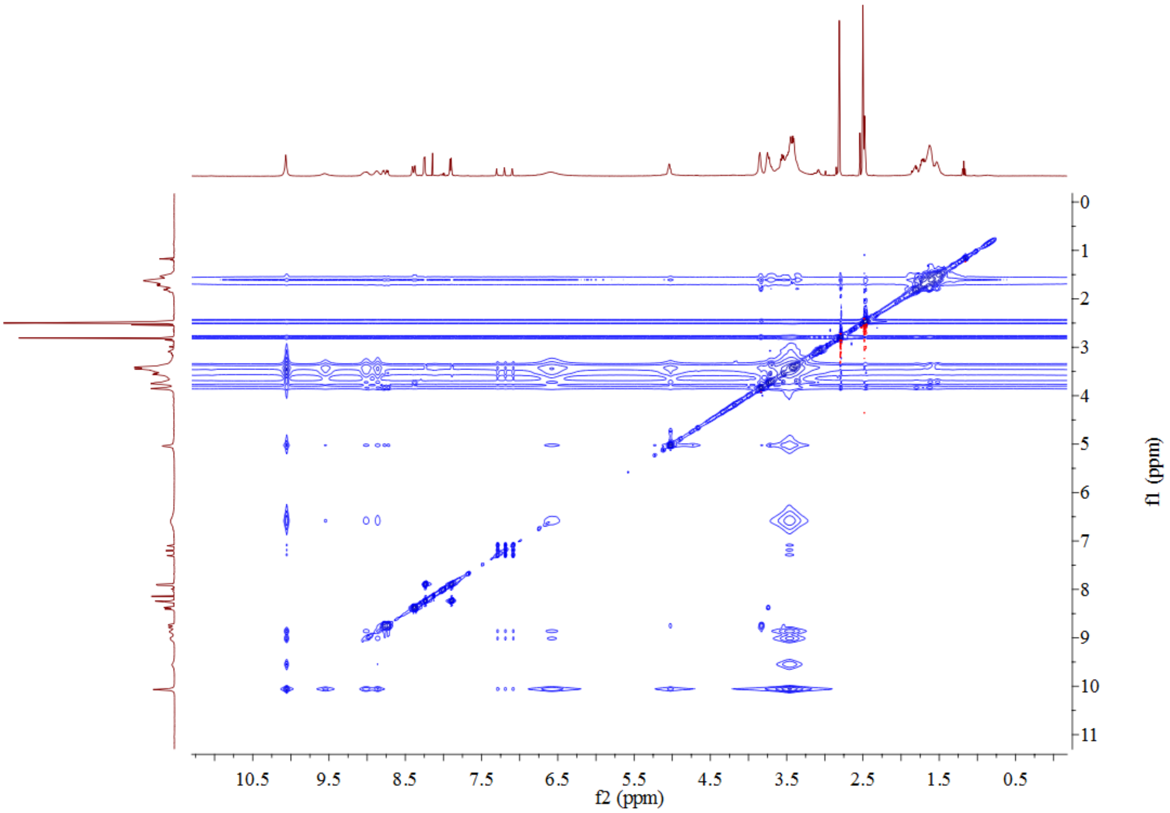

Figure S12. NOESY spectrum of albisporachelin in DMSO-*d6*.

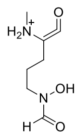

y_1

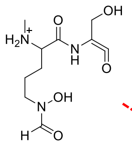

y_2

[M+H]+

	m/z (Da)	Centroid mass (Da)	Resolution	Centroid intensity	Intensity sum	Is Mono-Isotopic
1	108.07871	108.07971	2.0469e4	5.3462	18.0769	Undefined
2	113.06941	113.06981	1.8468e4	8.0308	27.6923	Undefined
3	114.05277	114.05362	2.1666e4	14.5615	48.6538	Undefined
4	127.08487	127.08529	2.2361e4	221.3077	740.6154	Undefined
5	129.09930	129.10081	2.4800e4	4.5846	15.2308	Undefined
6	131.07945	131.08023	2.3592e4	10.4769	34.7308	Undefined
7	145.09586	145.09580	2.3518e4	356.9538	1241.1923	Undefined
8	159.07347	159.07488	2.1642e4	4.8038	16.5385	Undefined
9	169.09528	169.09570	2.5846e4	8.3846	28.2308	Undefined
10	173.08997	173.09051	2.3765e4	34.5308	116.1923	Undefined
11	186.12163	186.12207	2.2460e4	4.6615	15.6923	Undefined
12	187.10523	187.10598	2.4969e4	71.4231	240.7308	Undefined
13	209.12517	209.12666	2.3415e4	11.5654	40.1923	Undefined
14	214.11587	214.11680	2.5716e4	25.9231	87.4231	Undefined
15	215.09979	215.10074	2.8173e4	10.7692	36.1538	Undefined
16	232.12576	232.12699	2.5741e4	335.2942	1156.7500	Undefined
17	242.11092	242.11138	2.5747e4	52.8769	184.5385	Undefined
18	242.14677	242.14755	2.4190e4	23.0077	80.1154	Undefined
19	257.15674	257.15849	2.5847e4	8.1769	28.1538	Undefined
20	260.12110	260.12162	2.6168e4	219.2692	761.7308	Undefined
21	264.09002	264.09067	2.7608e4	14.6000	50.1154	Undefined
22	270.14140	270.14214	2.5134e4	35.0538	119.8077	Undefined
23	275.16523	275.16881	2.3379e4	6.3769	21.4615	Undefined
24	276.08902	276.09005	2.8915e4	20.5000	70.5385	Undefined
25	285.15133	285.15278	2.6404e4	126.3885	436.8846	Undefined
26	287.16639	287.16796	2.9352e4	4.0462	14.6923	Undefined
27	303.16310	303.16306	2.6427e4	327.8173	1138.0962	Undefined
28	344.18659	344.18883	3.0744e4	4.6423	15.1154	Undefined
29	372.18525	372.18336	2.6456e4	20.7885	75.0769	Undefined
30	390.19367	390.19372	2.6228e4	109.1077	380.3846	Undefined
31	400.17627	400.17835	2.4800e4	10.7231	35.8462	Undefined
32	544.26401	544.26597	2.8195e4	27.8269	97.9615	Undefined
33	562.27532	562.27620	2.9589e4	48.2538	180.0769	Undefined
34	562.36001	562.35945	2.4995e4	7.8500	27.0000	Undefined

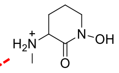

b_3

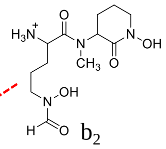

b_2

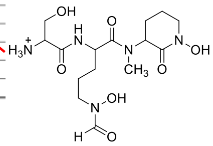

b_1

Figure S13. Observed main fragments list of compound **1** during MS^2 fragmentation experiments.

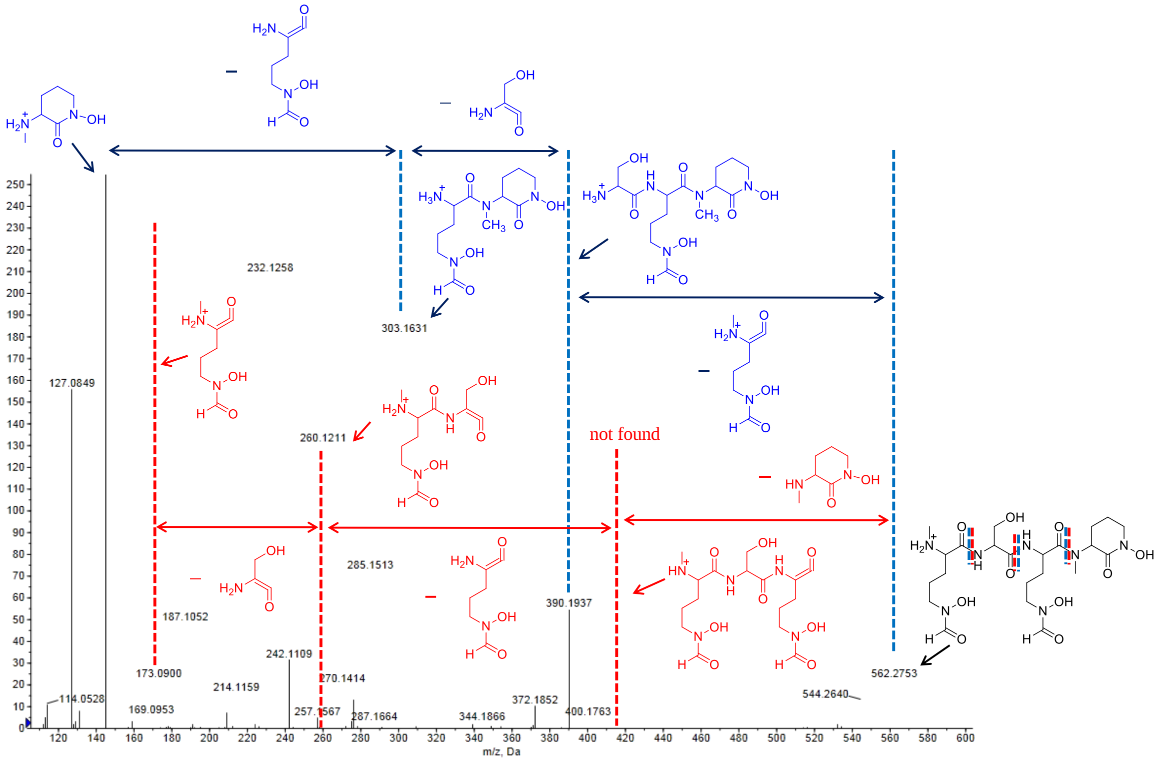

Figure S14. Main fragments observed during MS² fragmentation experiments and assignment of the molecular ion peak.

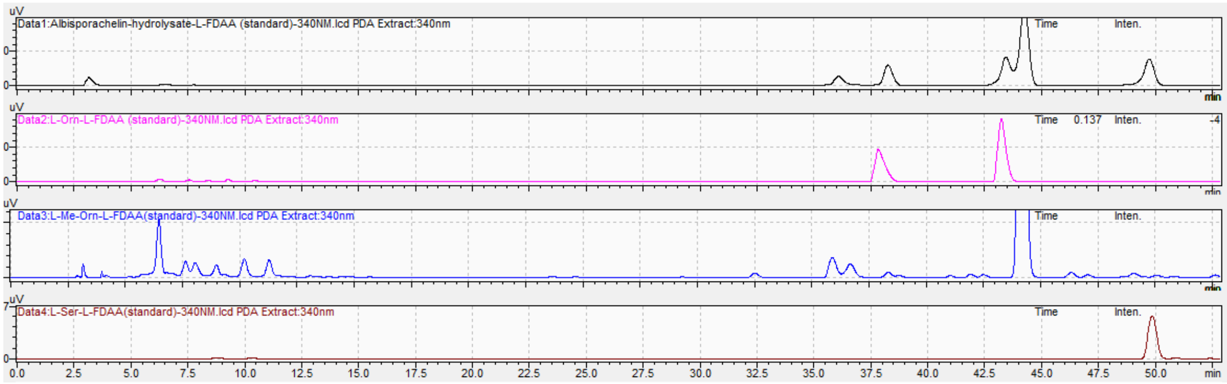

Figure S15. HPLC trace of FDAA-derivatized albisporachelin hydrolysate with standard. Trace A is albisporachelin hydrolysate; trace B is standard L-Orn-L-FDAA; trace C is standard L-Me-Orn-L-FDAA; trace D is standard L-Ser-L-FDAA.

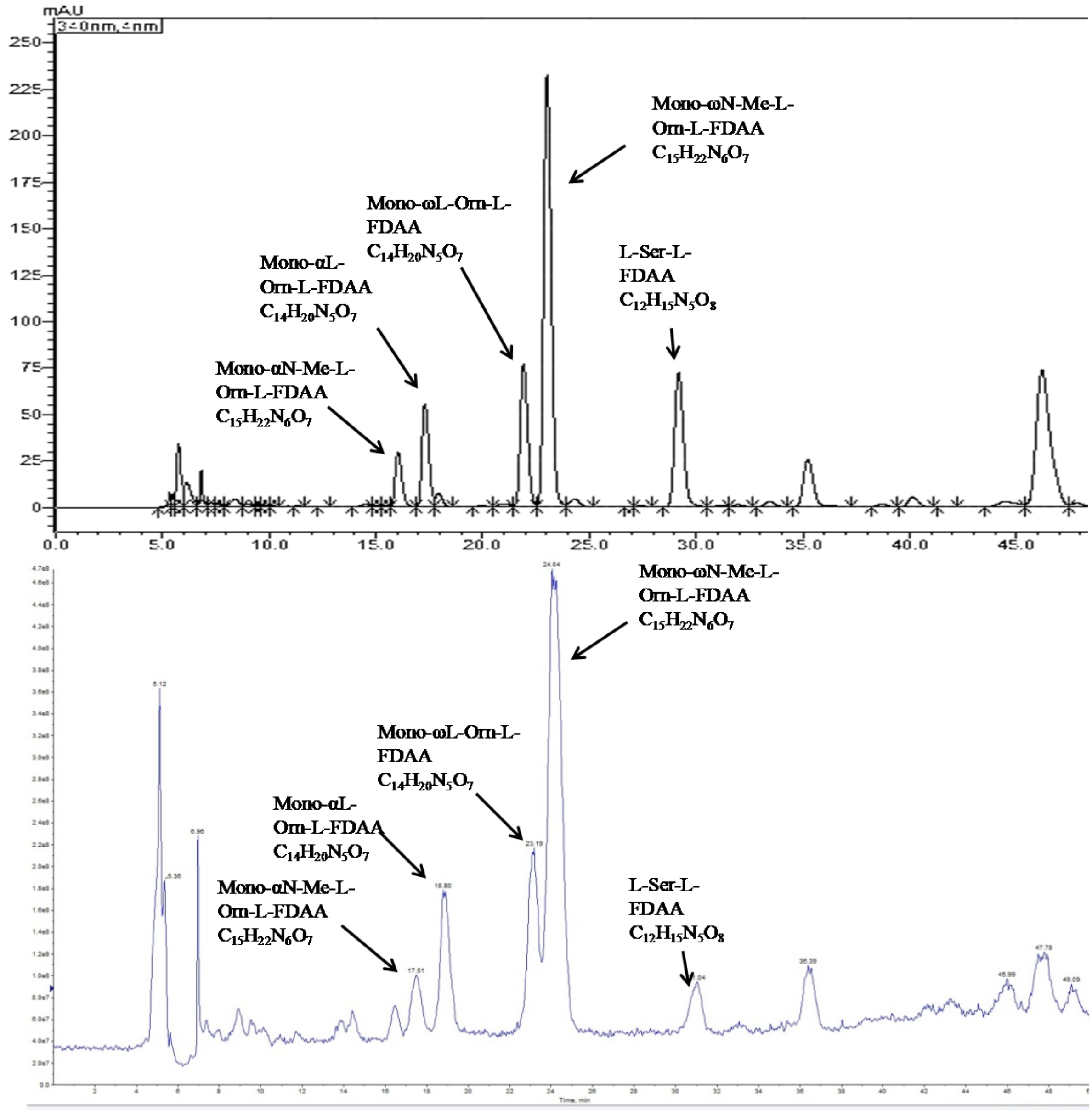

Figure S16. LC-MS-traces of FDAA-derivatized albisporachelin hydrolysate.

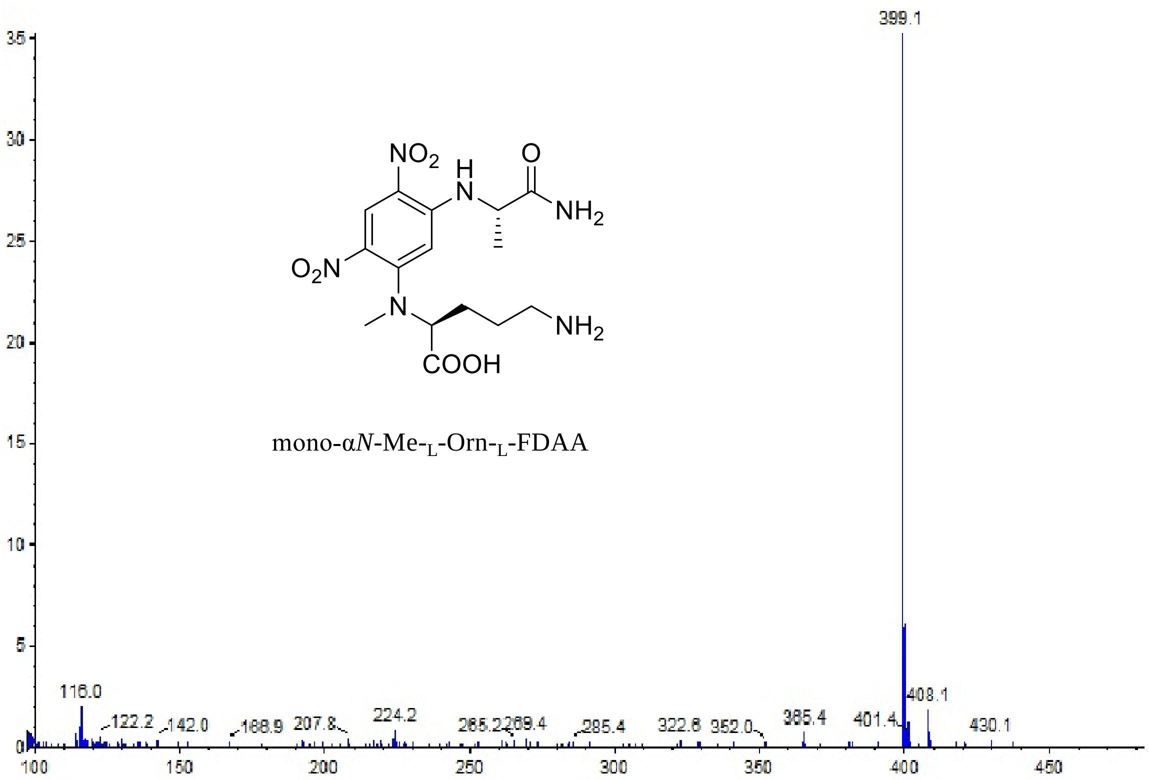

Figure S17. MS spectrum of mono-αN-Me-L-Orn-L-FDAA in albisporachelin hydrolysate.

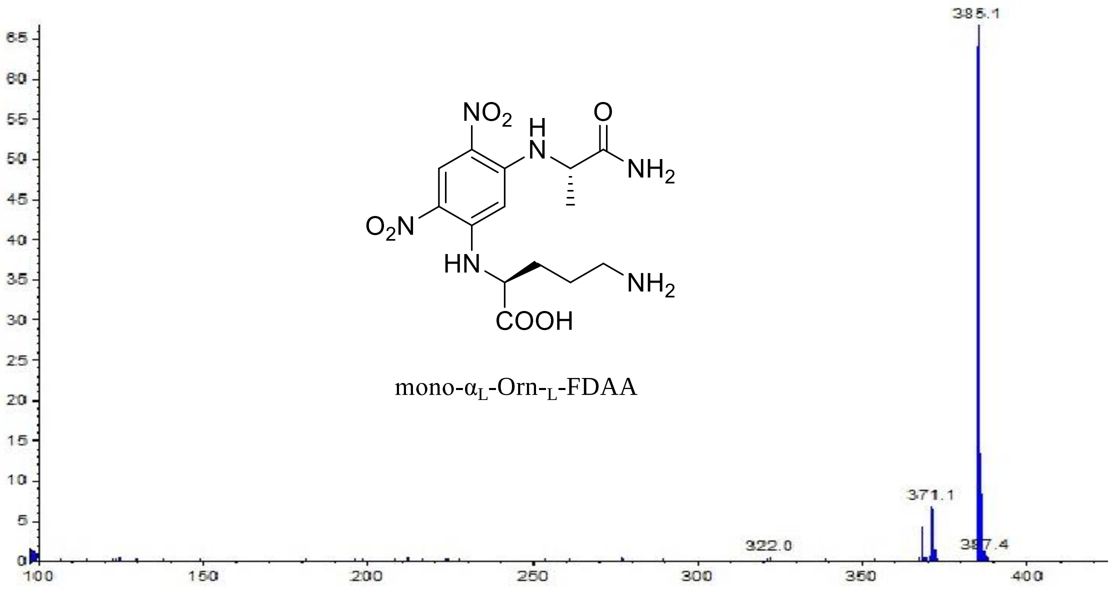

Figure S18. MS spectrum of mono-αL-Orn-L-FDAA in albisporachelin hydrolysate.

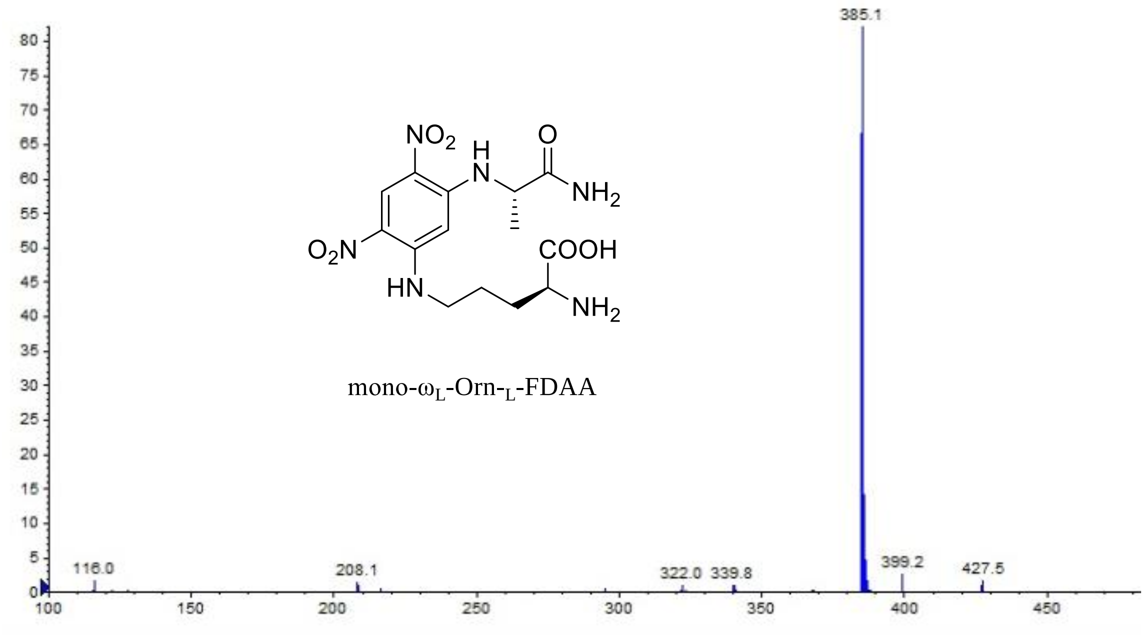

Figure S19. MS spectrum of mono-ωL-Orn-L-FDAA in albisporachelin hydrolysate.

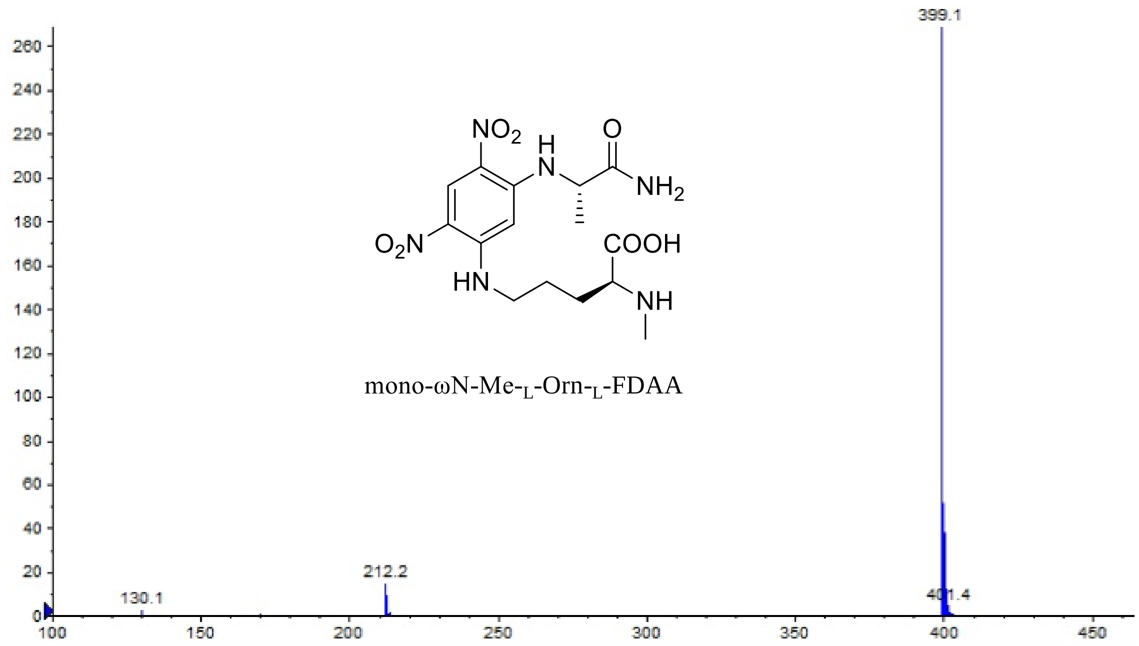

Figure S20. MS spectrum of mono-ωN-Me-L-Orn-L-FDAA in albisporachelin hydrolysate.

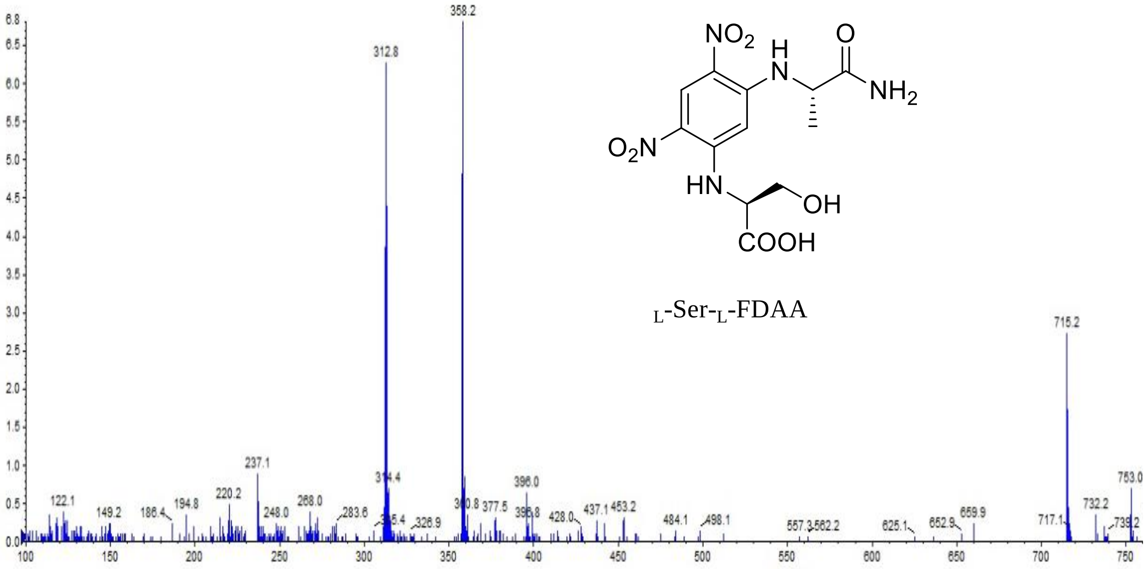

Figure S21. MS spectrum of L-Ser-L-FDAA in albisporachelin hydrolysate.

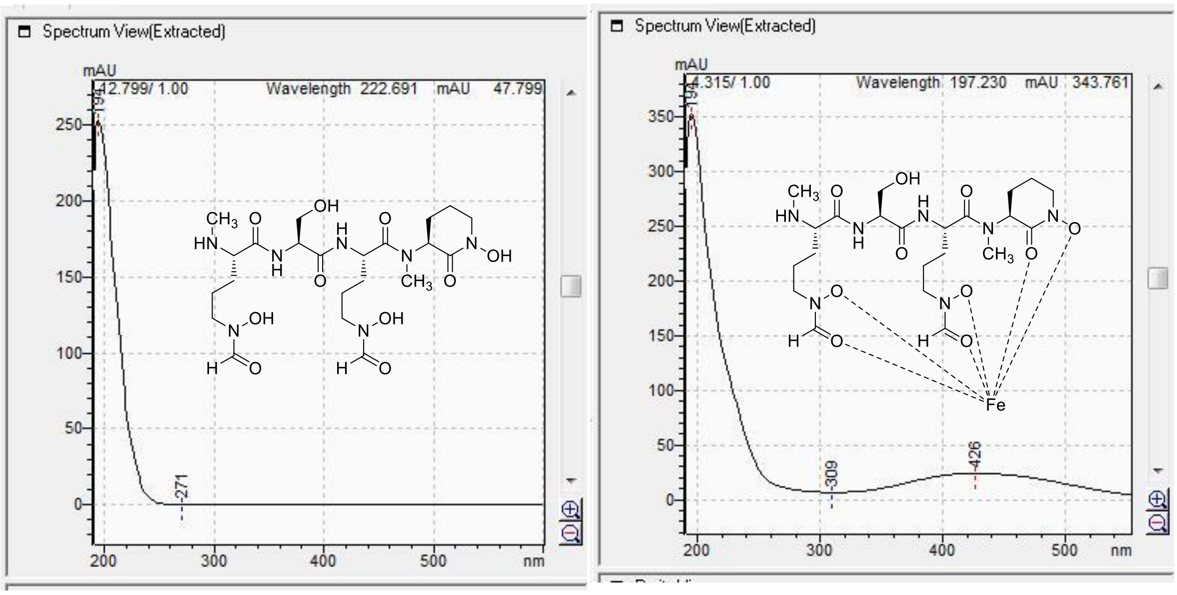

Figure S22. UV-visible spectra of albisporachelin and ferric complex of albisporachelin.
